# Progress toward Elimination of Trachoma as a Public Health Problem in Seven Localities in the Republic of Sudan: Results from Population-Based Surveys

**DOI:** 10.4269/ajtmh.19-0530

**Published:** 2019-10-07

**Authors:** Angelia M. Sanders, Zeinab Abdalla, Belgesa E. Elshafie, Mazin Elsanosi, Andrew W. Nute, Nabil Aziz, E. Kelly Callahan, Scott D. Nash

**Affiliations:** 1 The Carter Center, Atlanta, Georgia;; 2 The Carter Center, Khartoum, Sudan;; 3 National Program for Prevention of Blindness, Federal Ministry of Health, Khartoum, Sudan

## Abstract

Trachoma is the leading cause of infectious blindness in the world. After baseline surveys demonstrated that Sudan was endemic for trachoma, the Sudan Federal Ministry of Health (FMOH) Trachoma Control Program conducted trachoma prevention and treatment interventions in endemic localities. The Sudan FMOH conducted population-based trachoma prevalence surveys between September 2016 and April 2017 in seven localities across five states of Sudan to document current trachoma prevalence estimates and measure water, sanitation, and hygiene (WASH) indicators. Children aged 1–9 years were examined for five clinical signs of trachoma, and participants of all ages were examined for trachomatous trichiasis (TT). A household questionnaire was administered to gather demographic and WASH-related information. The prevalence of trachomatous inflammation-follicular (TF) in children aged 1–9 years ranged from 0.4% (95% CI: 0.1–1.1%) to 6.4% (95% CI: 3.3–11.9%). Trachomatous trichiasis in those aged 15 years and older ranged from 0.1% (95% CI: 0.0–0.6%) to a high of 4.4% (95% CI: 2.1–9.1%). Of seven localities surveyed, four localities had achieved the elimination threshold of less than 5% TF in children aged 1–9 years. Six localities still required interventions to achieve less than 0.2% TT in those aged 15 years and older. The presence of latrine ranged from a low of 10.8% (95% CI: 5.2–21.1%) to 88.4% (CI: 81.5–93.0%) and clean face among children ranged between 69.5% (95% CI: 63.5–75.0%) and 87.5% (95% CI: 81.2–91.9%). These results demonstrate that Sudan is within reach of eliminating trachoma as a public health problem.

## INTRODUCTION

Trachoma is the leading cause of infectious blindness and is responsible for approximately 1.9 million people being blind or visually impaired.^1^ Countries such as Sudan have worked hard to reduce the risk of blindness from trachoma through the implementation of the World Health Organization’s (WHO) SAFE (Surgery for those who have the advanced stage of the disease, Antibiotic distribution to treat infection, and Facial cleanliness and Environmental improvement initiatives to decrease transmission) strategy.^2^ For a country to be considered as having eliminated trachoma as a public health problem, trachomatous inflammation-follicular (TF) must be less than 5% in children aged 1–9 years for a period of at least 2 years and trachomatous trichiasis (TT), the advanced stage of the disease, must be less than either 0.2% in those aged 15 years and older or 0.1% in the total population.^3^

Between 2006 and 2010, trachoma baseline surveys were conducted across Sudan to determine whether Sudan was endemic for trachoma.^4^ Trachomatous inflammation-follicular in children aged 1–9 years was above the WHO elimination threshold in 15 localities (the equivalent of a district). The prevalence of TT was above the threshold among adults aged 15 years and older in 48 localities. Further baseline mapping conducted between 2014 and 2015 in Darfur and Khartoum documented TF in children aged 1–9 years above the threshold in 11 localities and TT above the threshold in 30 localities, all in the Darfur region.^5^ These two sets of baseline surveys demonstrated that of 131 localities surveyed, 26 localities required investment in water, sanitation, and hygiene (WASH) programs and between one to three rounds of mass drug administration (MDA) with azithromycin to reduce infection. Seventy-eight localities required TT surgical interventions.

Following these baseline surveys, the Sudan Trachoma Control Program began implementation of the SAFE strategy in endemic localities. Seven of these endemic localities, El Fashaga, El Quraisha, and Baladyat el Gedarif localities in Gedarif state; Sawakin locality, Red Sea state; El Dinder locality, Sinnar state; Gaissan locality, Blue Nile state; and Abu Jebaiha locality, South Kordofan state, implemented SAFE activities; however, the degree to which the activities were implemented and the number of years they were implemented varied between localities. For example, when the program began MDA distributions in the seven localities, there were not clear WHO guidelines regarding how many rounds of MDA should be conducted in districts with TF prevalence between 5.0% and 9.9%; therefore, some localities received more rounds than the others (Table 1). In addition, in Abu Jebaiha locality, South Kordofan state and Gaissan locality, Blue Nile state, only one round of MDA was conducted as internal displacement and security concerns between 2012 and 2015 made it difficult for the national program to consistently access the area. For all MDAs conducted in the seven localities, administrative coverage was reported at greater than 80% of the targeted population.

**Table 1:** Trachomatous inflammation-follicular baseline survey results and rounds of mass drug administration for seven localities in Sudan

State	Locality	Type of survey*	Trachomatous inflammation-follicular prevalence in children aged 1–9 years	Rounds of MDA after baseline survey‡
Red Sea	Sawakin	Baseline	6.5	3
Gedarif	El Fashaga	Baseline	6.1	2
Gedarif	El Quraisha	Baseline	8.5	3
Sinnar	El Dinder	Baseline	8.5	2
Blue Nile	Gaissan†	Baseline	17.4	1
South Kordofan	Abu Jebaiha†	Baseline	6.1	1
Gedarif	Baladyat el Gedarif	Baseline	5.9	3

MDA = mass drug administration.

* Baseline surveys were conducted between 2006 and 2010. Results were published in 2011.^4^

† Localities that experienced insecurity which limited programmatic access.

‡ MDA rounds were conducted between the years 2011 and 2016.

All seven localities implemented surgical campaigns at the locality level, and surgeries were performed by trained ophthalmologists and ophthalmic residents. All identified TT patients were offered surgery at the time of identification during surgical campaigns. Those who refused surgery were counseled on its benefits. Health education messaging was conducted through the distribution of posters, flipcharts, and leaflets, and via radio messaging, television, and loudspeaker announcements during all MDA and surgical campaigns. In addition, all seven localities implemented a primary and secondary school–based trachoma health education program developed by the Sudan FMOH National Trachoma Control Program, the Federal Ministry of Education National Centre for Curriculum and Educational Research department, and The Carter Center.^6^ The National Trachoma Control Program does not directly provide latrines to households but advocates to the United Nations Children’s Fund (UNICEF) and the Water and Environmental Sanitation department for water and latrine provision in endemic localities.

After multiple years of SAFE interventions, in 2016 and 2017, the Sudan National Trachoma Control Program conducted prevalence surveys in seven localities to generate more current estimates of the trachoma prevalence among children aged 1–9 years and adults aged 15 years and older. These surveys also collected information on household demographics and WASH indicators. The results of these surveys provide important programmatic data for subsequent trachoma interventions in the country as Sudan seeks to eliminate trachoma as a public health problem.

## METHODS

### Setting.

Administratively, Sudan is divided into states, localities, administrative units, and villages. Localities are the equivalent of a district and are the level at which trachoma activities are typically implemented. Population-based surveys were conducted in five localities in 2016 (Sawakin, September; El Fashaga, November; El Quraisha, November; El Dinder, November; and Gaissan, December), whereas two localities were surveyed in 2017 (Abu Jebaiha in February and Baladyat el Gedarif in April).

### Sampling.

To estimate TF prevalence among children aged 1–9 years with 95% confidence, we assumed TF prevalence in this age-group of 3% ± 2% precision and a design effect of 3.0, and, thus, would require 837 children.^7^ We further assumed a nonresponse rate of 20%, 4.7 individuals per household, and that children aged 1–9 years make up 35% of the population.^4^ Based on these assumptions, we targeted 1,004 children per locality. To achieve this sample size, we surveyed 25 clusters (villages) and within each cluster we surveyed 25 households.

For all seven surveyed localities, the same multistage cluster random sampling method was used. In the first stage, clusters were selected from a geographically ordered list using a probability proportional to estimated population size method for each locality based on village population estimates provided by the State Ministry of Health Expanded Program on Immunization. Villages with a population < 250 persons were excluded because of concerns of meeting the minimum sample size requirements, and towns with a population > 5,000 persons were excluded from the sampling frame as they were considered as urban centers.^8–10^ In the second stage, the village community leaders created a list of households by household father’s name, and individual households were grouped into 5-household “segments” and numbered. The numbers were written on individual pieces of paper and placed in a bowl for random selection. Five segments were then chosen by a village leader.

During the surveying process, several protocol exceptions occurred. In El Quraisha locality, the data from three clusters surveyed were lost and, therefore, were unable to be included as part of that locality’s analysis. In Sawakin locality, the number of children per household was lower than that previously assumed. To ensure the minimum target population was reached, 10 additional segments were added in the last cluster, bringing the total number of segments for that cluster to 15.

### Data collection.

All data were collected electronically on Samsung Galaxy tablet computers loaded with custom-built survey software.^11^ Teams comprised a trachoma grader, a data recorder, and a driver. One supervisor was responsible for two teams, with all surveys having five teams. All residents of selected households were enumerated, regardless of their presence and/or willingness to be examined. All present and consented children aged 1–9 years were observed for all five signs of trachoma as defined by the WHO-simplified grading scheme using a 2.5× loupe and a flashlight.^12^ They were also assessed for a clean face, defined as the absence of ocular or nasal discharge. Participants aged 1 year and older were examined for TT. If TT was found, the eyelid was flipped and examined for trachomatous scarring (TS). At the end of each day, survey teams made one follow-up attempt to return to homes to examine children who were absent during the examination process. Households that were empty were not replaced by another household. Any participant found to have TF or trachomatous inflammation-intense (TI) was provided with antibiotics according to national guidelines, and those with TT were registered and counseled to have TT surgery during the next scheduled surgical campaign in their locality.

### Training of data collectors.

Data recorders were selected from the endemic states in which the trachoma program worked. All recorders underwent a 2-day training on how to use electronic tablets to collect data, conduct interviews, and randomly select households and individuals to be interviewed following the standard protocol. All data recorders were required to pass an examination on their data collection skills to participate on the survey team. Selected ophthalmologists and ophthalmic residents working in Sudan were trained as trachoma “graders.” Grader training consisted of in-class and field practice using the WHO-simplified grading system.^12^ Each grader was required to pass both a pre-created in-class slide test that included all five stages of trachoma and a field reliability examination with a score of 84% agreement and ≥ 0.70 kappa score against the consensus grade of the grader trainers to join the survey teams.

### Household interview.

Before the survey, the household questionnaire was translated into Arabic. Structured interviews with adult household respondents were conducted at each selected household to assess demographic and household characteristics. If more than one adult resident was present, special preference was given to female caregivers because they are usually the primary caregivers to children and are predominantly responsible for fetching water and performing household chores. In households where a latrine was present, recorders and/or graders directly observed the presence of the latrine and asked the respondent if they used the latrine. Respondents were asked questions regarding household socioeconomic indicators such as mobile phone, livestock, and radio ownership; levels of education; how often children’s faces were cleaned each day; time to collect water (< 30 minutes, 30–60 minutes, and > 60 minutes); and access to an improved water source. For purposes of analysis, an improved water source was defined according to the WHO/UNICEF Joint Monitoring Programme for Water Supply and Sanitation, and included protected dug well, protected spring, public tap, borehole, or piped water into dwelling.^13^

### Data entry and analysis.

All statistical analyses were conducted using Stata 13.1 (StataCorp LP [http://www.stata.com]). Sampling weights were calculated as the inverse of the probability of selection at both stages of sampling. Taylor linearization through *svy* survey procedures in Stata 13.1 was used to calculate CIs, taking into account the multilevel structure of the sampling. Post-stratification weighting using 5-year age–sex bands from the survey census population was also used when estimating the prevalence of TT among participants aged 15 years and older to account for systematic absence among older males in this population.^14^ The absence of males was perceived to be an issue because women carry an increased burden of TT compared with men.^15^ Trachomatous trichiasis unknown to the health system was defined as anyone who had not had surgery and had not refused surgery for at least one eye presenting the clinical sign of TT. All reported percentages with CIs were weighted.

### Ethical considerations.

The Sudan Federal Ministry of Health and Emory University Internal Review Board (IRB) (IRB 079-2006) provided ethical clearance. Because of logistical constraints of written consent forms and illiteracy among the population, IRB approval was obtained for verbal informed consent to be collected from all participants and recorded electronically. For those younger than 16 years, verbal consent from a parent or guardian was obtained. Participants were free to withdraw consent at any time without consequence.

## RESULTS

**Figure 1.**
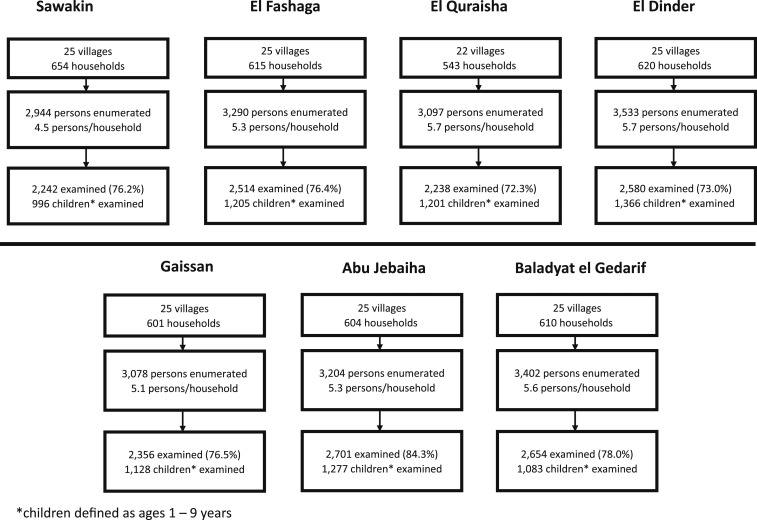
Survey population by locality, Sudan, 2016–2017.

The survey was undertaken in seven localities, and the sample comprised 172 clusters from which a total of 22,548 individuals in 4,247 households were enumerated (Figure 1). Of those enumerated, 18,323 were present at the time of the survey (a response rate of 81.3%). Among present household members, 17,285 (94.3%) consented to the examination and were included in the analysis. A total of 8,256 (93.0%) children aged 1–9 years were present and examined of 8,880 children enumerated. In the examined population with complete gender data, 57.6% were female and 42.4% were male. Among children aged 1–9 years, 49.0% were female and 51.0% were male. The mean age of all survey respondents was 17.4 years (SD ± 0.13 years).

Indicators of water, sanitation, and hygiene varied across all the seven localities (Table 2). Prevalence of an improved primary source of water ranged from 10.7% (95% CI: 3.3–29.3%) in Sawakin to 79.4% (95% CI: 60.8–90.5%) in El Fashaga locality. Households that take less than 30 minutes roundtrip to collect water ranged from 46.0% (95% CI: 29.7–63.1%) in Abu Jebaiha to 90.4% (95% CI: 70.3–97.4%) in El Dinder locality. Overall, the prevalence of directly observed clean face among children aged 1–9 years ranged between 69.5% (95% CI: 63.5–75.0%) in Gaissan and 87.5% (95% CI: 81.2–91.9%) in El Fashaga locality. Presence of latrine ranged from a low of 10.8% (95% CI: 5.2–21.1%) in Abu Jebaiha to 88.4% (95% CI: 81.5–93.0%) in Gaissan locality. Mobile phone ownership was higher than 50% in all the surveyed localities, ranging from a low of 51.5% (95% CI: 34.0–86.7%) in El Dinder to 88.6% (95% CI: 82.8–92.7%) in Baladyat el Gedarif, the state capital of Gedarif state.

**Table 2:** Individual and household characteristics by locality, Sudan, 2016–2017

Characteristics	Sawakin, % (95% CI)	El Fashaga, % (95% CI)	El Quraisha, % (95% CI)	El Dinder, % (95% CI)	Gaissan, % (95% CI)	Abu Jebaiha, % (95% CI)	Baladyat el Gedarif, % (95% CI)
Individual							
Children aged 1–9 years with clean face (observed)	85.9 (81.8–89.3)	87.5 (81.2–91.9)	78.5 (72.9–83.3)	85.8 (75.6–92.2)	69.5 (63.5–75.0)	75.3 (68.5–81.1)	85.4 (79.9–89.6)
Household							
Caregivers washing children’s faces							
Every few days or never	2.0 (0.5–8.3)	0.0	0.0	0.7 (0.2–2.2)	1.1 (0.2–5.5)	0.5 (0.2–1.8)	0.1 (0.0–0.9)
Once a day	21.8 (11.9–36.5)	15.6 (5.3–37.7)	2.6 (1.3–4.8)	14.6 (9.0–22.9)	12.8 (6.5–23.6)	48.9 (27.4–70.7)	2.9 (0.9–8.4)
Twice a day	20.0 (13.7–28.4)	19.4 (15.3–24.3)	23.7 (18.7–29.5)	32.4 (23.5–42.7)	25.5 (18.7–33.8)	12.8 (6.9–22.4)	6.0 (3.7–9.4)
More than twice a day	56.2 (41.5–69.8)	65.0 (46.9–79.7)	73.8 (67.6–79.1)	52.4 (37.4–67.0)	60.7 (47.9–72.1)	37.9 (21.3–57.9)	91.1 (84.7–94.9)
Presence of latrine (observed)	57.3 (41.6–71.6)	67.4 (48.0–82.2)	37.9 (26.0–51.4)	29.5 (16.6–46.7)	88.4 (81.5–93.0)	10.8 (5.2–21.1)	77.7 (65.6–86.4)
Improved primary source of water	10.7 (3.3–29.3)	79.4 (60.8–90.5)	55.6 (41.1–69.2)	70.3 (44.7–87.4)	32.1 (17.5–51.4)	70.2 (45.2–87.1)	77.0 (59.5–88.4)
Time to collect water (minutes)							
< 30	82.4 (63.3–92.7)	87.0 (66.0–95.8)	82.7 (67.9–91.5)	90.4 (70.3–97.4)	76.8 (58.2–88.7)	46.0 (29.7–63.1)	68.6 (53.8–80.4)
30–60	6.0 (2.4–14.3)	9.1 (2.2–31.2)	17.3 (8.5–32.1)	5.7 (0.8–31.0)	12.3 (4.8–28.0)	42.5 (27.0–59.7)	20.4 (11.4–33.7)
> 60	11.7 (4.0–29.8)	3.9 (0.6–21.4)	0.0	3.9 (0.9–15.0)	10.9 (3.7–28.0)	11.6 (3.1–34.6)	11.0 (4.6–24.1)
Livestock ownership	54.3 (40.8–67.3)	45.8 (31.5–60.9)	50.9 (44.5–57.3)	70.5 (53.3–83.3)	43.8 (36.4–51.6)	77.8 (67.6–85.4)	26.2 (21.0–32.2)
Radio ownership	14.1 (6.5–28.0)	20.2 (13.9–28.3)	32.6 (25.3–40.9)	26.0 (16.7–38.2)	33.5 (24.3–44.1)	40.4 (28.4–53.6)	24.4 (15.4–36.3)
Mobile phone ownership	66.7 (54.5–77.0)	74.6 (61.5–84.4)	72.7 (64.2–79.8)	51.5 (34.0–68.7)	70.5 (61.5–78.1)	64.3 (53.4–73.9)	88.6 (82.8–92.7)
Any adult education	84.1 (66.6–93.4)	69.8 (52.0–83.1)	84.9 (77.9–90.0)	49.9 (34.5–65.3)	90.8 (86.5–93.8)	66.0 (54.4–76.0)	95.8 (92.5–97.7)

**Figure 2.**
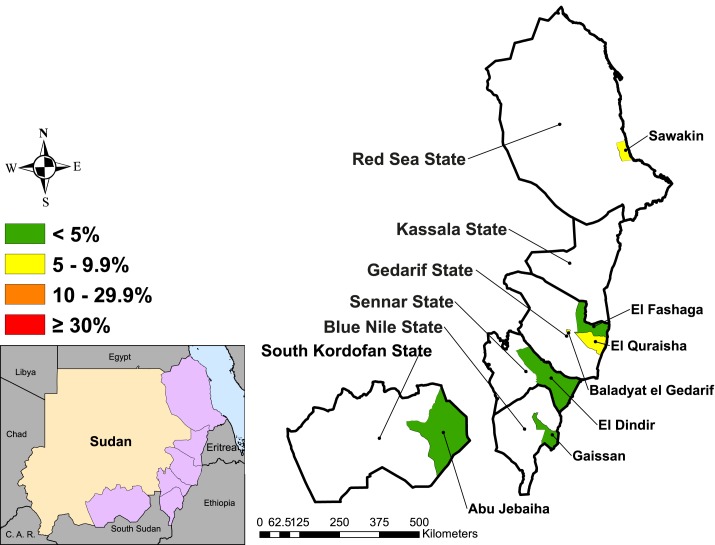
Prevalence of trachomatous inflammation-follicular in children aged 1–9 years in seven localities in Sudan, 2016–2017. This figure appears in color at www.ajtmh.org.

**Figure 3.**
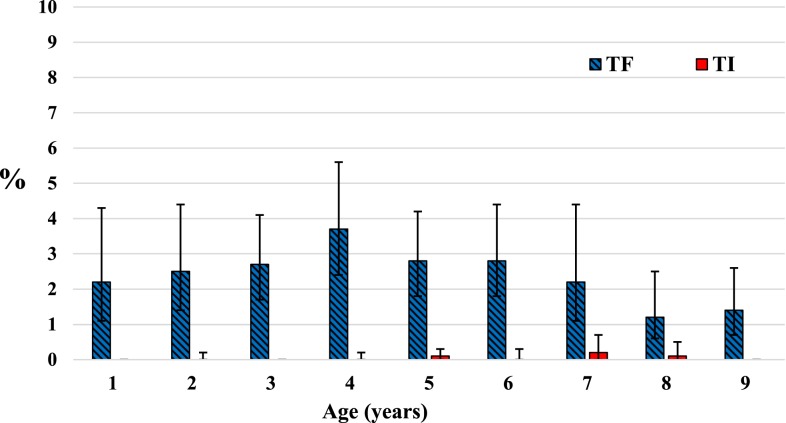
Age-specific prevalence of trachomatous inflammation-follicular and trachomatous inflammation-intense among children aged 1–9 years in seven localities in Sudan, 2016–2017. This figure appears in color at www.ajtmh.org.

The prevalence of TF among children aged 1–9 years ranged from 0.4% (95% CI: 0.1–1.1%) in El Fashaga to a high of 6.4% (95% CI: 3.3–11.9%) in El Quraisha locality (Table 3). Three of the localities had greater than 5% TF among children aged 1–9 years (Figure 2). A detectable level of TI was found only in El Quraisha at 0.4% (95% CI: 0.2–1.0%). Overall, the prevalence of TF among children aged 1–9 years did not statistically significantly differ between males 2.6% (95% CI: 1.9–3.7%) and females 2.3% (95% CI: 1.6–3.4%), *P* = 0.412. Within this age-group, TF point estimates were higher among younger children (< 7 years) than among children aged between 7 and 9 years (Figure 3).

**Table 3:** Prevalence of clinical signs of trachoma in seven localities in Sudan, 2016–2017

Clinical sign	Sawakin, % (95% CI)	El Fashaga, % (95% CI)	El Quraisha, % (95% CI)	El Dinder, % (95% CI)	Gaissan, % (95% CI)	Abu Jebaiha, % (95% CI)	Baladyat el Gedarif, % (95% CI)
Trachomatous inflammation-follicular, age 1–9 years	6.2 (3.6–10.4)	0.4 (0.1–1.1)	6.4 (3.3–11.9)	1.7 (0.9–3.5)	2.6 (1.2–5.9)	1.0 (0.4–2.5)	5.4 (2.4–11.8)
TI, age 1–9 years	0.0	0.0	0.4 (0.2–1.0)	0.0	0.0	0.0	0.0
TT, all ages	0.3 (0.1–0.8)	0.3 (0.1–0.7)	0.1 (0.0–0.3)	0.1 (0.1–0.4)	2.0 (0.9–4.4)	0.0 (0.0–0.2)	0.2 (0.1–0.5)
TT, ages ≥ 15 years	0.6 (0.2–1.9)	0.7 (0.3–1.8)	0.4 (0.1–1.0)	0.4 (0.2–1.0)	4.4 (2.1–9.1)	0.1 (0.0–0.6)	0.5 (0.2–1.2)
TT, ages ≥ 15 years*	0.4 (0.2–1.3)	0.6 (0.2–1.4)	0.3 (0.1–0.8)	0.4 (0.2–1.3)	4.3 (2.0–9.2)	0.1 (0.0–0.4)	0.5 (0.2–1.2)
TT, ages ≥ 15 years, unknown to health system*	0.2 (0.1–0.6)	0.3 (0.1–0.9)	0.3 (0.1–0.8)	0.4 (0.2–1.3)	2.2 (1.0–4.8)	0.0	0.4 (0.1–1.0)
TT with TS, ages ≥ 15 years*	0.1 (0.0–0.4)	0.1 (0.0–0.9)	0.1 (0.0–0.5)	0.1 (0.0–0.7)	1.8 (0.7–4.7)	0.0	0.3 (0.1–1.0)
Corneal opacity, ages ≥ 15 years	0.0 (0.0–0.3)	0.0	0.0	0.0	0.0 (0.0–0.3)	0.0	0.0

TI = trachomatous inflammation-intense; TS = trachomatous scarring; TT = trachomatous trichiasis.

* Post-stratification weighting with 5-year age–sex bands applied.

**Figure 4.**
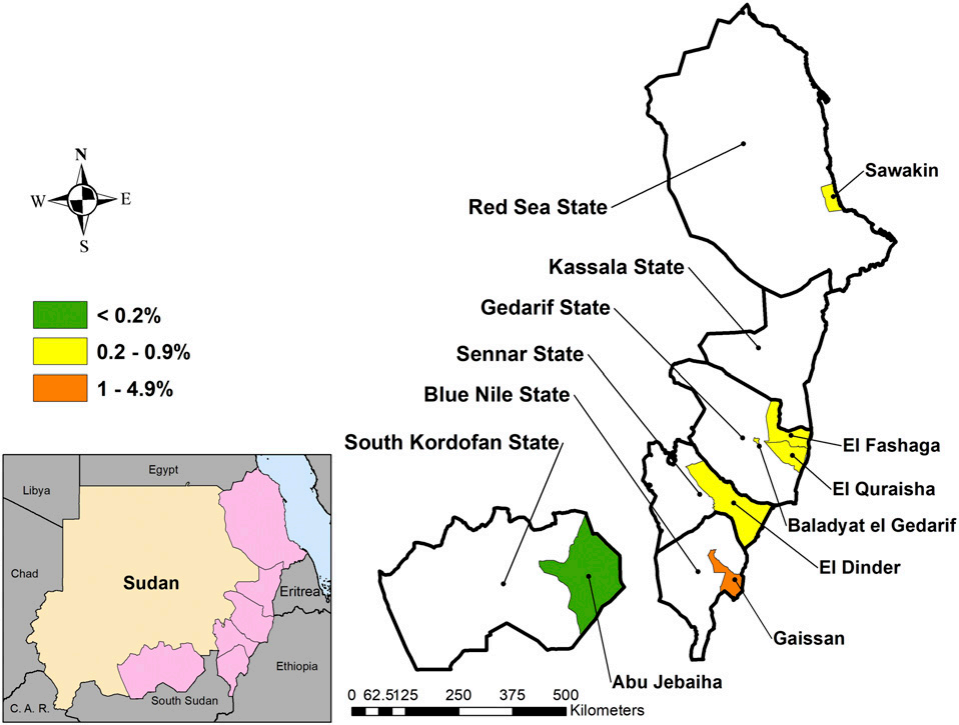
Prevalence of trachomatous trichiasis in adults aged 15 years and older in seven localities in Sudan, 2016–2017. This figure appears in color at www.ajtmh.org.

The prevalence of TT in adults aged 15 years and older ranged from 0.0% (95% CI: 0.0–0.4%) in Abu Jebaiha to 4.3% (95% CI: 2.0–9.2%) in Gaissan locality (Figure 4). When examining TT cases in those aged 15 years and older that are unknown to the health system, the TT prevalence ranged from 0.0% (95% CI: 0.0–0.0%) in Abu Jebaiha to 2.2% (95% CI: 1.0–4.8%) in Gaissan. Six of the seven localities, therefore, exceeded the WHO target of < 0.2% TT in those aged 15 years and older unknown to the health system. Among participants aged 15 years and older with TT and available data (*n* = 47), 32.9% (95% CI: 16.9–54.1%) reported having had trichiasis surgery and 50.5% (95% CI: 32.2–68.7%) were observed by the grader as having signs of epilation. Overall, 50% of TT cases had concurrent signs of TS. Corneal opacity was found only in Sawakin (four cases) and Gaissan (one case) localities.

## DISCUSSION

The results of these prevalence surveys demonstrated that Sudan is within reach of its goal of eliminating trachoma as a public health problem in these seven localities. Four localities were below the TF elimination threshold among children aged 1–9 years, and the remaining three localities had a prevalence lower than 7% TF. Trachomatous inflammation-intense was detected only among children aged 1–9 years in one locality, El Quraisha at 0.4%, suggesting that the prevalence of *Chlamydia trachomatis* infection was most likely very low in all the seven localities.^16^ Six of the seven localities were still above the WHO TT unknown to the health system elimination threshold in those aged 15 years and older; however, five of these localities ranged between 0.2% and 0.4%. Only one locality, Gaissan, had greater than 1.0% TT. With concerted effort over the next few years, particularly in identifying and operating on TT cases, Sudan should be able to join the growing ranks of formerly trachoma-endemic countries.

The purpose of these seven surveys was to provide a current TF and TT prevalence figure with which to plan future interventions. Moving forward, different programmatic interventions are needed in different localities. Only one locality, Abu Jebaiha, reached the WHO elimination targets for both TF and TT. The other six localities, therefore, require continued implementation of different aspects of the SAFE strategy. Although the point estimates for three localities were above the 5% TF threshold, it is likely that trachoma is no longer a public health problem among children in any of the seven localities, particularly because no TI was found in all but one locality. However, because evidence shows that one round of MDA provided in districts with TF between 5% and 9.9% can decrease TF prevalence less than 5%,^17^ we recommend that three localities (Sawakin, El Quraisha, and Baladyat el Gedarif) conduct one additional round of MDA followed by a survey to measure impact. Four localities (El Fashaga, El Dinder, Gaissan, and Abu Jebaiha) have met the TF elimination target and no longer require MDA. After at least 2 years of no MDA, these four localities should conduct a surveillance survey to confirm that TF has remained less than 5% and that TT has reduced less than or remained less than 0.2% in adults aged 15 years and older.^3^ All but Abu Jebeiha locality will require surgical campaigns to reach the TT threshold. These surgical campaigns should be conducted alongside the development of a sustainable TT service delivery mechanism that ensures future incident cases of TT can be managed by the health system. All the seven localities should continue implementing health education activities that focus on facial cleanliness and the building and use of latrines.

The TF results from these seven surveys demonstrated that the longitudinal trends following SAFE interventions were not homogenous across the seven localities. For example, Gaissan locality in Blue Nile state, a locality that experienced population displacement for years because of insecurity, had 17.4% TF in children aged 1–9 years at baseline, received one round of MDA, and then had a 2.6% TF prevalence in this survey. This contrasts with Baladyat el Gedarif locality in Gedarif state which had 5.9% TF at baseline, three rounds of MDA, and a documented TF prevalence above the threshold (5.4%) at impact survey. Baladyat el Gedarif, the state capital of Gedarif state, had a higher proportion of children with clean face (85%), higher access to improved primary sources of water (77%), and a higher percentage of adults reporting adult education (96%) than Gaissain locality. The different trajectories in these localities highlight the challenges of comparing prevalence trends across localities following implementation of SAFE interventions. It is unclear why the prevalence decreased dramatically within one locality and not the other and what, if any, the SAFE strategy played in these results. Ultimately, we know that Sudan was not hyperendemic for trachoma at baseline and that the results of these surveys demonstrate that Sudan has either met or is close to meeting elimination thresholds.^4,5^

The results of these surveys should be taken in light of several limitations. Slight protocol deviations occurred in two of the localities; however, the targeted sample size of children was reached in all localities. In regard to TT, the CIs were wide, given that the survey was designed to estimate TF in children. Trachomatous trichiasis prevalence should, therefore, be interpreted as a range and used as an estimate when planning surgical campaigns. In the six localities that still required surgical interventions, three (El Quraisha, El Dinder, and Baladyat el Gedarif) had TT prevalence estimates that were nearly identical between known and unknown to the health system, which highlights that more needs to be carried out to identify and offer services to those with TT. In the remaining three localities (Sawakin, El Fashaga, and Gaissan), when unknown to the health system was taken into consideration, the prevalence decreased by half. In the 47 identified TT cases where data were available, 50% were observed as having signs of epilation. Previous research in Ethiopia found comparable results between patients with minor TT who epilate versus those receiving surgery.^18^ Therefore, the national program should consider adopting an epilation program for individuals who either decline surgery or are unable to access surgical treatment immediately. Given that 78 localities had greater than 0.2% TT in adults aged 15 years and older at baseline, and that only one of these 78 localities is documented as reducing TT below the 0.2% TT threshold, achieving and documenting TT reduction below elimination thresholds will remain one of Sudan’s biggest challenges in reaching elimination targets. Developing a systematic case-finding approach and epilation program may work to help overcome this remaining hurdle.

## CONCLUSION

The Sudan Trachoma Control Program has conducted years of programmatic interventions to eliminate trachoma as a public health problem by 2020. These population-based prevalence surveys demonstrated that in seven localities documented as being endemic for trachoma during baseline surveys, four have reached the TF elimination threshold, with one of these four localities also achieving the TT threshold. Six localities will require continued surgical interventions to reach the TT threshold. With continued commitment to implement the full SAFE strategy, the Sudan Trachoma Control Program is on target to meet its goal of eliminating trachoma as a public health problem in the remaining six localities.
